# A Two-Step Synthesis of Porous Nitrogen-Doped Graphene for Electrochemical Capacitors

**DOI:** 10.3390/ijms252212297

**Published:** 2024-11-15

**Authors:** Jiahao Li, Zhenjia Wu, Rong Huang, Anbang Ge, Jie Ying

**Affiliations:** School of Chemical Engineering and Technology, Sun Yat-sen University, Zhuhai 519082, China; lijh255@mail2.sysu.edu.cn (J.L.); wuzhj58@mail2.sysu.edu.cn (Z.W.); huangr279@mail2.sysu.edu.cn (R.H.); geanb@mail2.sysu.edu.cn (A.G.)

**Keywords:** porous nitrogen-doped graphene, petroleum asphalt, electrochemical capacitors

## Abstract

Porous nitrogen-doped graphene (PNG) materials with high conductivity, high surface area, and chemical stability have displayed superior performance in electrochemical capacitors. However, previously reported methods for fabricating PNG render the processes expensive, hard to control, limited in production, and unsafe as well, thus largely restricting their practical applications. Herein, we present a facile two-step calcination method to prepare PNG using petroleum asphalt as the carbon source to provide the original three-dimensional porous structure directly and using environmentally friendly and high nitrogen content urea as the nitrogen source without adding any etching agent. The porous structure in PNG can largely increase its specific surface area, and the introduction of nitrogen atoms can effectively increase the degree of defects and improve the wettability of PNG. As a result, PNG displays a high specific capacitance of 157 F g^−1^ at a current density of 1 A g^−1^ and cycling stability while maintaining 98.68% initial capacitance after 10,000 cycles.

## 1. Introduction

The development of high-power-density storage devices is regarded as indispensable because of the booming market of mobile and immobile electronic devices [1,2,3]. Electrochemical capacitors, otherwise known as supercapacitors, which offer high power density, long cycle life, and rapid charge–discharge capacity, have attracted extensive attention [4,5,6,7]. It is widely recognized that the performance of electrochemical capacitors is mainly decided by the electrode materials. Thus, the design and fabrication of high-performance electrode materials can contribute to enhancing the properties of electrochemical capacitors. Recently, a series of carbon materials with high conductivity has been investigated as efficient electrode materials for electrochemical capacitors [8,9,10,11,12,13]. Among them, porous nitrogen-doped graphene (PNG) materials display high surface area and chemical stability and thus have high electrochemical performance such as capacitance, rate capability, and cycling stability [14,15,16,17,18,19].

At present, PNG can be produced either using graphene oxide through self-assembly [20,21,22] and 3D printing [23,24,25] or by way of chemical vapor deposition [26,27,28]. In these configurations, pores are created between nanosheets by means of cross-linking gelation or a template drive, endowing the graphene frameworks with diverse structures and multiple functions. Moreover, it has been confirmed that introducing heteroatom dopants such as nitrogen into graphene can further modulate their electronic properties and electrochemical performance [15,29,30]. However, these methods for manufacturing PNG render the processes expensive, hard to control, limited in production, and unsafe as well, thus severely restricting their practical applications [31,32]. Therefore, the development of a simple, low-cost, and environmentally friendly approach for the fabrication of PNG is of considerable significance.

As a low-value by-product in petroleum processing, petroleum asphalt has attracted much attention because of its low cost and large quantity (over 25 million tons per year) [19,33]. The content of polycyclic aromatic hydrocarbons in petroleum asphalt is rich, and the content of ash is low, so the carbon material produced by carbonization has excellent porosity and ideal electrical conductivity [34,35]. Herein, we report the fabrication of PNG through a two-step calcination method using petroleum asphalt as a carbon source and urea as a nitrogen source without adding any etching agent. The porous structure effectively increases the specific surface area of PNG and promotes the transfer of electrolytic ions. In addition, the introduction of nitrogen atoms increases the degree of defects and improves the wettability of the material. Benefiting from this, PNG has superior specific capacitance and cycle stability, proving its good electrochemical properties as an electrode material for electrochemical capacitors.

## 2. Results and Discussion

**Figure 1:**
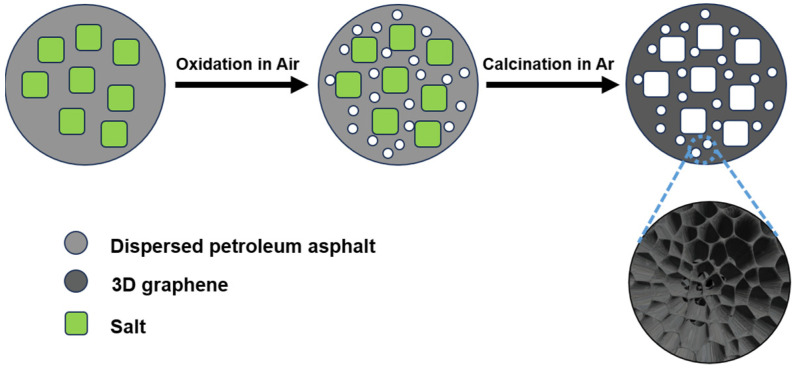
Scheme illustration of synthesis of PNG-x.

The overall synthetic procedure of PNG-x (x represents the amount of urea added) is displayed in Figure 1. Asphalt, urea, and mixed salt are oxidized in the air after ball milling, and the unstable components in the mixture are decomposed into CO_2_ and CO gas molecules, thus obtaining pre-PNG with mesoporous and macroporous. After high-temperature carbonization, the cross-linked oxygen-containing functional groups between the carbon layers can be removed, thereby expanding the carbon layer spacing. Then the mixed salt is removed with deionizing water, and finally, PNG-x is obtained.

**Figure 2:**
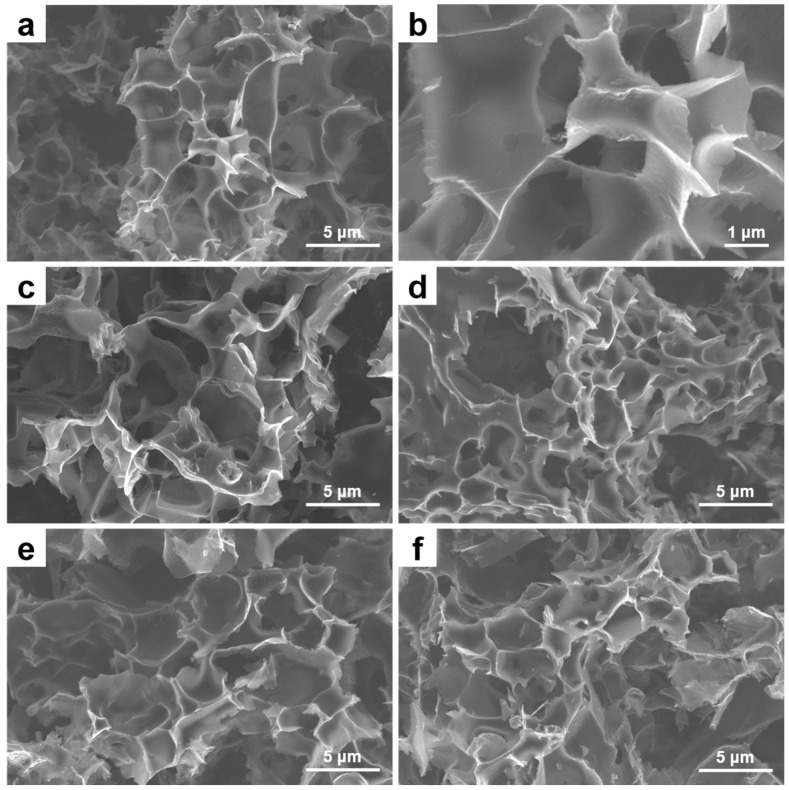
SEM images of (**a**,**b**) PNG-0.6, (**c**) PNG-0, (**d**) PNG-1.2, (**e**) PNG-1.5, (**f**) PNG-2.0.

**Figure 3:**
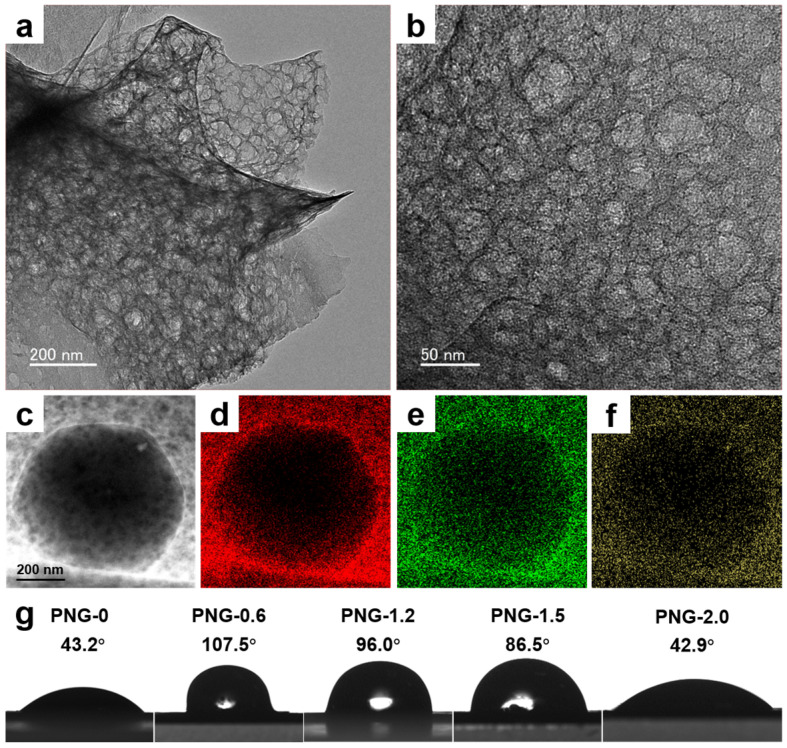
(**a**,**b**) TEM images of PNG-0.6, (**c**) HAADF-STEM images of PNG-0.6, (**d**–**f**) EDS elemental mappings of C, N, and O, (**g**) water drops on PNG-x.

The morphologies of PNG-x are shown in Figure 2a–f. It can be seen that PNG-x has an obvious porous network structure, and with the increase in urea content, the morphology of PNG-x still maintains a porous structure. This proves that the two-step oxidation carbonization method can be effectively applied to the production of porous structures. In order to further confirm the mesostructure and element distribution of PNG-x, PNG-0.6 is characterized by transmission electron microscopy (TEM). Figure 3a,b are TEM images of PNG-0.6 at different magnifications. As can be seen from Figure 3a, PNG-0.6 has a clear three-dimensional porous structure. In addition, the lamellar surface of PNG-0.6 is rich in mesoporous pores (Figure 3b). Figure 3c presents the high-angle annular dark-field scanning TEM (HAADF-STEM) image of a part of PNG-0.6, and the corresponding elemental mapping images in Figure 3d–f indicate that the elements C, N, and O are homogeneously distributed throughout PNG-0.6, revealing that nitrogen obtained from urea decomposition can be effectively doped into graphene. It is worth noting that the three-dimensional porous structure of PNG-x has little effect on wettability, and PNG-x exhibits hydrophobicity only at suitable nitrogen doping levels (Figure 3g). Among them, PNG-0.6 exhibits hydrophobicity, which can avoid the adsorption of water and minimize the impact of water on the performance of the capacitor [36].

**Figure 4:**
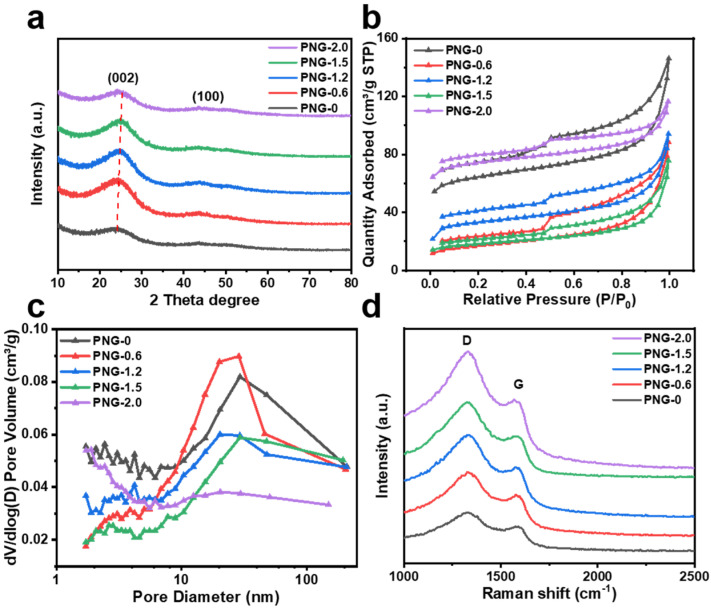
(**a**) XRD patterns of PNG-x, (**b**) N_2_ adsorption and desorption isotherm curves of PNG-x, (**c**) diameter distribution curves of PNG-x, (**d**) Raman spectra of PNG-x.

The crystalline structure of all the samples is determined using X-ray diffraction (XRD) (Figure 4a). For all samples, two broad peaks can be observed on the XRD pattern, corresponding to the (002) and (100) crystal faces of the carbon material [18,37,38]. The diffraction angle 2θ of PNG-0.6 at the (002) plane is around 24.4°, and the average layer spacing of PNG-0.6 can be calculated as 0.358 nm according to the Bragg equation. With the increase in urea content, the diffraction peak of PNG-x at (002) plane gradually moves to the higher angle, which proves that the increase in urea content will reduce the layer spacing of PNG-x.

The nitrogen adsorption/desorption isotherms of the samples are shown in Figure 4b. A typical type IV isotherm characteristic accompanied by a hysteresis loop is observed, indicating that there are a large number of mesopores in PNG-x [39,40]. As indicated in Table 1, the specific surface areas of the PNG-0, PNG-0.6, PNG-1.2, PNG-1.5, and PNG-2.0 samples, which are calculated based on the Brunauer–Emmett–Teller (BET) method, are 242, 61, 128, 67, and 283 m^3^ g^−1^ respectively. The pore volumes are 0.226, 0.137, 0.146, 0.117, and 0.180 cm^3^ g^−1^, respectively. The pore size of PNG-x calculated using the Barrett–Joyner–Halenda (BJH) method is mainly distributed in the mesoporous region (Figure 4c). In addition, the results in Table 1 show that adding a small amount of urea can significantly reduce the content of micropores, leading to a decrease in surface area and pore volume. With the increase in urea content, the micropore content gradually increases, and the surface area and pore volume of PNG-x will increase significantly.

Raman spectroscope is a unique, non-destructive technique to investigate the structure properties of carbon materials. Figure 4d is the Raman spectrogram of PNG-x, from which peak D near 1330 cm^−1^ and peak G near 1570 cm^−1^ are observed. As shown in Table 2, the I_D_/I_G_ ratio of PNG-x gradually increased with the increase in urea content. The higher the I_D_/I_G_ ratio, the higher the defect degree of PNG-x and the lower the graphitization degree, indicating that the incorporation of nitrogen atoms provided defect sites [41,42,43]. Moreover, the I_D_/I_G_ ratio of PNG-x supplemented with urea is all greater than PNG-0 (1.08), which is largely due to the destruction of local symmetry by nitrogen atom doping, resulting in an increase in D-band intensity.

**Figure 5:**
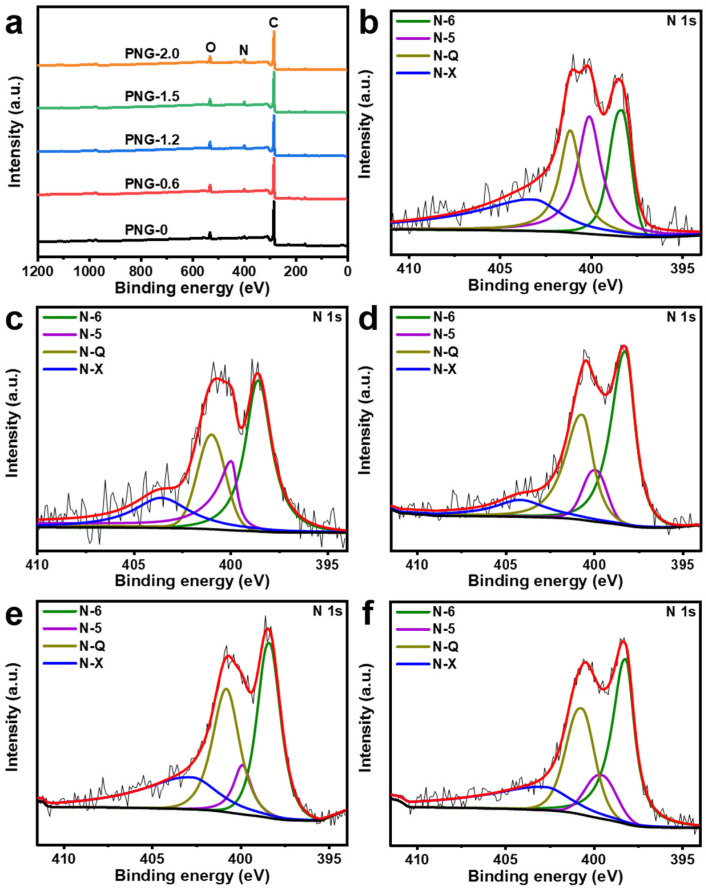
(**a**) XPS survey spectra of PNG-x, XPS high-resolution N 1s spectra of (**b**) PNG-0.6, (**c**) PNG-0, (**d**) PNG-1.2, (**e**) PNG-1.6, and (**f**) PNG-2.0.

X-ray photoelectron spectroscopy (XPS) is further employed to analyze the surface compositions of PNG-x. The full XPS spectra in Figure 5a clarified that all the PNG-x are composed of C, N, and O. The N 1s high-resolution peak of the PNG-0.6 in Figure 5b can be modeled into four independent peaks of 389.4 eV, 400.1 eV, 401.1 eV, and 403.1 eV, corresponding to the characteristic peaks of pyridinic-N (N-6), pyrrolic-N (N-5), graphitic-N (N-Q), and oxidized-N (N-X), respectively [44,45,46,47,48]. In addition, the N 1s high-resolution spectra of other samples, as shown in Figure 5c–f, can be convolved into four peaks of N-6, N-5, N-Q, and N-x. The nitrogen content of PNG-x increased from 4.18 at% to 8.96 at% with the increase in urea content (Table 3). Meanwhile, with the increase in defect nitrogen content (PNG-0 < PNG-0.6 < PNG-1.5 < PNG-1.2 < PNG-2.0), the specific surface area of PNG decreases first and then increases. This may be because the three-dimensional porous structure of PNG is first partially destroyed with the increase in urea, resulting in a decrease in the specific surface area. Then, the urea content continues to increase, which forms part of the porous carbon material, resulting in an increase in the specific surface area. Supercapacitors are classified into electrical double-layer capacitors and pseudocapacitors based on their charge storage mechanisms [49]. Among these nitrogen functional groups, N-6 and N-5 are thought to contribute more to specific capacitors by introducing pseudocapacitors, while N-Q can effectively improve the conductivity of carbon materials by promoting electron transfer, which means that PNG-0.6 has good supercapacitor performance [14].

**Figure 6:**
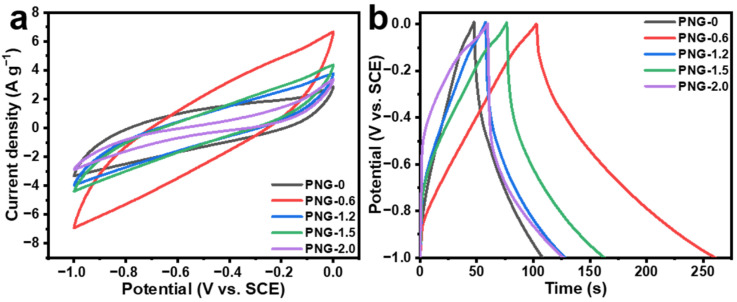
(**a**) CV curves of PNG-x at 100 mV s^−1^, (**b**) GCD curves of PNG-x at 1 A g^−1^.

The electrochemical properties of PNG-x samples were examined in an alkaline medium. As shown in Figure 6a, cyclic voltammetry (CV) curves of different samples at a scanning rate of 100 mV s^−1^ are presented. All samples showed an approximate spindle shape, indicating the existence of electric double-layer capacitors. It is worth noting that PNG-0.6 has the largest total area. It is widely known that the specific capacitance is in proportion to the area of the CV curve, thus indicating that less nitrogen doping will increase the overall capacitance.

The specific capacitance values of the PNG-x computed from galvanostatic charge–discharge (GCD) curves (refer to Figure 6b) were 60 F g^−1^ (PNG-0), 157 F g^−1^ (PNG-0.6), 69 F g^−1^ (PNG-1.2), 89 F g^−1^ (PNG-1.5), and 66 F g^−1^ (PNG-2.0), respectively. It can be seen that the nitrogen-doped sample has a higher specific capacitance than the sample without urea. PNG-0.6 has a high specific capacitance mainly for the following two reasons: (1) pyridinic-N and pyrrolic-N can enhance electrochemical reactivity, and (2) the rich pore structure is beneficial for improving the contact area between the electrode and the electrolyte and reducing the resistance to transfer of electrolytic ions [50,51]. Although the surface area of PNG-1.2 and PNG-2.0 is much larger than that of PNG-0.6, their high nitrogen doping actually hinders electron transfer and reduces capacitance. PNG-1.5 also has a lower surface area than PNG-0.6, lacking enough space to transfer electrolytic ions.

**Figure 7:**
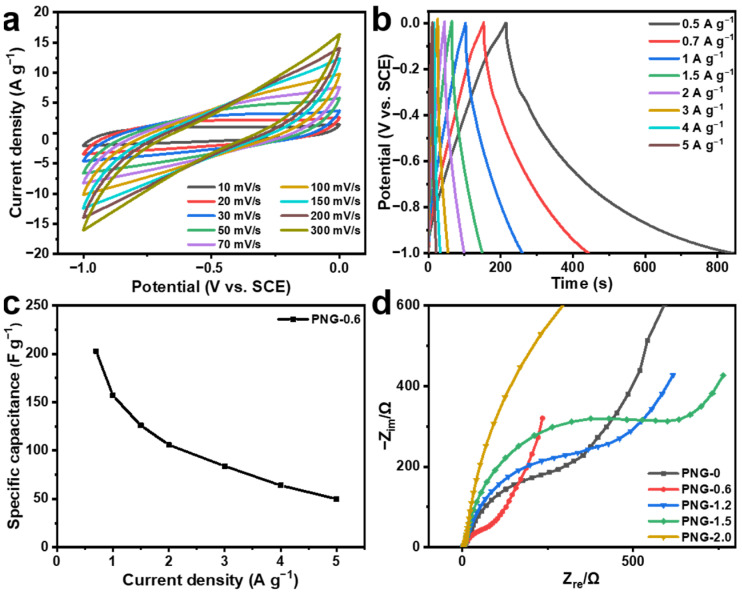
(**a**) CV curves at different scan rates for the PNG-0.6. (**b**) GCD curves at different current densities for PNG-0.6, (**c**) the specific capacitance versus current density for PNG-0.6, (**d**) Nyquist plots of PNG-x.

Figure 7a presents the CV curves of the PNG-0.6 at different scan rates varying from 10 to 300 mV s^−1^. With the increase in the scan rate, the CV loop maintains its spindle shape, suggesting that very little polarization occurs because of the high electronic conductivity of PNG-0.6. The GCD curves of PNG-0.6 are shown in Figure 7b. The curves are nearly triangular in shape, featuring slight curvature and no obvious potential drop at a current density ranging from 0.5 and 5 A g^−1^, which indicates its outstanding electrochemical reversibility and rapid I–V response. Figure 7c plots the relationship between specific capacitance and current density of PNG-0.6. As the current density increases, the specific capacitance gradually decreases, which might be attributed to the limited diffusion of electrolyte ions. In addition, when the current density rises from 1 A g^−1^ to 5 A g^−1^, about 34% of the specific capacitance scale remains unchanged, indicating that PNG-0.6 has excellent rate capability for electrochemical capacitors. This may be related to the porous structure and excellent electronic conductivity of PNG-0.6 [52].

To further explore the electrochemical behavior of the PNG-x sample, electrochemical impedance spectroscopy (EIS) tests were carried out at an open-circuit voltage in the frequency range from 10^−2^ Hz to 10^5^ Hz. As shown in Figure 7d, all samples except PNG-2.0 exhibit vertical slopes in the low-frequency region, suggesting that the sample has good capacitance and low ion diffusion resistivity at the electrode/electrolyte interface. Moreover, the charge transfer impedance of PNG-0.6 is considerably lower than that of other samples, indicating that proper nitrogen doping can efficiently enhance the electrochemical properties of the material. However, too much nitrogen atom doping will lead to a decrease in resistivity and not enough to achieve fast charge transfer kinetics.

**Figure 8:**
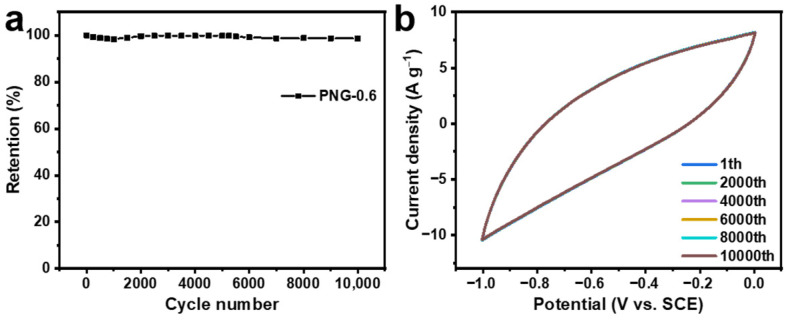
(**a**) Cycling stability of PNG-0.6, (**b**) The first, 2000th, 4000th, 6000th, 8000th, 10,000th CV curves of the PNG-0.6 at 200 mV s^−1^.

Cycling stability performance is a highly significant parameter for electrochemical capacitors. As demonstrated in Figure 8a, after 1000 cycles, the specific capacitance of PNG-0.6 began to increase, reaching a maximum of 99.92% of the initial specific capacity (3000 cycles). It may be that during the cycle, some of the graphitized carbon in PNG-0.6 degenerates to a more disordered degree, exposing more active sites and thus contributing to better contact between the electrode and the electrolyte [53]. And after 10,000 cycles, it can still retain 98.68% of the initial specific capacitance. The results show that PNG-0.6 has excellent cyclic stability. Moreover, the first, the 2000th, 4000th, 6000th, 8000th, and 10,000th CV curves of PNG-0.6 obtained at 200 mV^−1^ are shown in Figure 8b. The CV curves are almost identical, which further indicates that PNG-0.6 is an excellent and stable electrochemical electrode material.

## 3. Materials and Methods

### 3.1. Materials

Petroleum asphalt was purchased from Wentian New Materials Tech. Co., Ltd., Huizhou, China. Liquid aromatic petrochemical by-products were purchased from China Railway Weiye Waterproof Material Technology Co., Ltd., Jinan, China. Urea (CH_4_N_2_O), sodium chloride (NaCl), potassium chloride (KCl), and potassium hydroxide (KOH) were purchased from Aladdin Industrial Corporation, Shanghai, China. Nafion (5 wt%) was purchased from Sigma-Aldrich, Shanghai, China.

### 3.2. Synthesis of PNG-x

PNG-x was synthesized by a two-step oxidation carbonization process. In a typical procedure, 1 g petroleum asphalt was dispersed with 10 mL liquid aromatic petrochemical by-products by ultrasonic for 20 min. The above mixture was ball-milled with 20 g NaCl: KCl (mass ratio 4.5:5.5) and x (x = 0, 0.6, 1.2, 1.5, 2.0) g urea to obtain a homogeneous solid mixture. Next, the mixture was heated at a heating rate of 5 °C min^−1^ to 300 °C in a muffle furnace under air atmosphere and maintained for 2 h to obtain pre-PNG. Then, the sample was calcined at a heating rate of 3 °C min^−1^ to 700 °C under Ar atmosphere and maintained for 2 h, after which the sample was naturally cooled to room temperature. Finally, after washing and drying, PNG-x was obtained.

### 3.3. Characterization

Field-emission scanning electron microscopy (FESEM) was conducted on Thermoscientific Apreo 2S HiVac (Waltham, MA, USA) at the acceleration voltage of 5 kV, and TEM studies were carried out on JEOL JEM-F200 (Tokyo Japan) at the acceleration voltage of 200 kV. XRD measurements were carried out on an Ultima IV X-ray diffractometer (Tokyo, Japan) using Cu Kα radiation (λ = 1.54056 Å) with a step width of 0.05° and step time of 0.5. The specific surface area for BET measurements was conducted using a Micromeritics ASAP 2460 system (Norcross, GA, USA). The pore size distribution of each sample was computed from the adsorption isotherm branch using the BJH method. Raman analysis was performed with a confocal Raman microscope (CRM) (Alpha300R, WITec GmbH, Ulm, Germany) equipped with a TEM single-frequency laser (λ = 633nm, laser power = 2 mW, WITec GmbH, Ulm, Germany). The laser light was focused through a 50× oil immersion objective onto the sample, and the backscattered Raman signal was directed through an optic multifiber (50 μm diameter) to a spectrometer (300 g·mm^−1^ grating) and detected by the CCD camera. On the selected areas on the sample every 0.5 μm a full wavenumber range (50–2940 cm^−1^) Raman spectrum was acquired with an integration time of 300 s. XPS analysis was conducted on a Thermo Scientific K-Alpha X-ray photoelectron spectrometer (Waltham, MA, USA). The N 1s spectra were acquired at photon energy (hv) of 1486.6 eV and analyzer pass energies (PE) of 50 eV. The survey spectra were acquired at photon energy (hv) of 1486.6 eV and analyzer pass energies (PE) of 150 eV.

### 3.4. Electrochemical Measurements

Electrochemical measurements were performed on Metrohm Autolab (Herisau, Switzerland) PGSTAT 302N using a three-electrode cell. A platinum foil of 4 cm^2^ was employed as a counter electrode, and a saturated calomel electrode (SCE) was utilized as a reference electrode. The slurry-coated nickel foam was used as the working electrode. To prepare the slurry, 10 mg PNG-x and 1 mg acetylene black were dispersed in a mixed solution containing 950 μL isopropanol and 50 μL nafion solution (5 wt%) under ultrasonic treatment for 30 min. In total, 100 μL of the aforementioned mixture was dropped onto nickel foam, and the coated area was approximately 1 cm × 1 cm. Subsequently, the nickel foam was left to dry overnight in the air. The mass loading of PNG-x on the nickel foam was maintained at 1 mg. All the electrochemical measurements, including CV, GCD, and EIS, were performed in 1 M KOH solution at room temperature. Generally, the CV tests were conducted at a scan rate ranging from 10 to 150 mV s^−1^ within a potential window of −1.0 V to 0 V (versus SCE). Meanwhile, the GCD measurements were performed at a current density ranging from 0.5 to 5 A g^−1^ with a potential window of −1.0 to 0 V (versus SCE). The specific capacitance of all the samples can be acquired by computing from GCD curves in accordance with the following equation:

$$C_{s}=\frac{∆t\;\times I}{∆V\;\times m}$$

where C_s_, Δt, I, ΔV, and m represent the specific capacitance, the discharge time, the discharge current, the potential charge during discharge, and the mass of the PNG-x, respectively.

## 4. Conclusions

In summary, a three-dimensional porous nitrogen-doped graphene with a macro–mesoporous structure has been successfully prepared by a facile two-step calcination strategy. The macro–mesoporous structure can effectively promote the transfer of electrolytic ions. Moreover, the incorporation of nitrogen atoms also boosts the electrochemical properties of the materials. Notably, PNG-0.6 has a specific capacitance of 157 F g^−1^ when the current density is 1 A g^−1^, which is significantly higher than that of the other samples. The initial capacitance of 98.68% can be maintained after 10,000 CV cycles, indicating that PNG-0.6 is a promising electrochemical material. Our approach provides an opportunity to design novel porous materials with low cost, high capacitance, and long-term stability in the electrochemistry field.

## Figures and Tables

**Table 1:** Specific surface area and pore volume of PNG-x.

Sample	S_BET_^a^ m^2^ g^−1^	V_t_^b^ cm^3^ g^−1^	V_m_^c^ cm^3^ g^−1^
PNG-0	242	0.226	0.083
PNG-0.6	61	0.137	0.011
PNG-1.2	128	0.146	0.038
PNG-1.5	67	0.117	0.018
PNG-2.0	283	0.180	0.105

^a^ BET-specific surface area; ^b^ total pore volume from adsorption isotherms at a relative pressure P/P_0_ of 0.99; ^c^ t-plot micropore volume.

**Table 2:** I_D_/I_G_ of PNG-x.

Sample	I_D_/I_G_^a^
PNG-0	1.08
PNG-0.6	1.13
PNG-1.2	1.17
PNG-1.5	1.19
PNG-2.0	1.23

^a^ The intense ratio of D band and G band from Raman spectra.

**Table 3:** Nitrogen content (at%) and nitrogen binding state in PNG-x obtained by XPS.

Sample	The Ratio of Different N Groups (at%)				
	Total	Pyridinic-N	Pyrrolic-N	Graphitic-N	Oxidized-N
PNG-0	4.18	1.63	0.85	0.96	0.74
PNG-0.6	5.44	1.20	1.63	1.28	1.33
PNG-1.2	8.05	3.68	0.97	2.53	0.87
PNG-1.5	8.42	3.14	0.76	2.55	1.97
PNG-2.0	8.96	3.65	1.06	2.48	1.77

## Data Availability

The original contributions presented in this study are included in the article. Further inquiries can be directed to the corresponding author.
